# Subject-independent classification of ADHD from pediatric EEG: a leakage-aware deep learning framework with interpretable neurophysiological mapping

**DOI:** 10.3389/fnins.2026.1925266

**Published:** 2026-10-06

**Authors:** M. Geethapriya, Kalyanaraman Pattabiraman

**Affiliations:** School of Computer Science and Engineering, Vellore Institute of Technology, Vellore, Tamil Nadu, India

**Keywords:** Attention-Deficit/Hyperactivity Disorder (ADHD), data leakage, deep learning, electroencephalography (EEG), explainable AI, pediatric neuroscience, SHAP, subject-independent validation

## Abstract

**Background:**

The use of electroencephalography (EEG) in automated screening of Attention-Deficit/Hyperactivity Disorder (ADHD) has created a large body of literature that suggests promising outcomes, with reported classification accuracies often above 90%. A major methodological issue suggests that a substantial part of these results are actually due to data leakage, whereby EEG epochs pertaining to the same subject end up in both training and testing sets, allowing classifiers to capitalize on features specific to the individual rather than on genuine disorder-related biomarkers.

**Methods:**

We assessed the performance of three classical machine learning algorithms, namely, Logistic Regression (LR), Random Forest (RF), and Support Vector Machine (SVM), as well as three deep learning models: Long Short-Term Memory (LSTM), Convolutional Neural Network (CNN), and CNN-BiLSTM, on a standard pediatric EEG dataset containing 121 children and 4,173 epochs. The performance of each model was compared in terms of both segment-wise cross-validation (Protocol A) and subject-wise cross-validation (without any data leakage, Protocol B).To evaluate neurotopographic plausibility without making unsupported causal assertions, we applied Deep SHapley Additive exPlanations (SHAP) to the best-generalizing architecture.

**Results:**

The performance discrepancy was substantial. Under segment-wise evaluation, the SVM operating on power spectral density (PSD) features reached 90.0% accuracy (95% CI: [89.1%, 90.9%], AUC = 0.96). However, the same model dropped to 65.6% (95% CI: [62.1%, 69.1%], AUC = 0.72) under subject-wise evaluation—a statistically significant 24.4 percentage point collapse (*p* < 0.001, Wilcoxon signed-rank). In contrast, deep architectures learning directly from raw EEG were resilient: the stand-alone CNN lost only 2.5 percentage points, achieving 72.7% accuracy (95% CI: [66.0%, 79.4%], AUC = 0.72, recall = 88.0%) on completely unseen participants. *Post-hoc* SHAP attribution identified the highest relative contributions in frontal (F3, Fz, F4) and central (Cz) electrodes, aligning with established ADHD neurophysiology.

**Conclusion:**

Hand-engineered spectral features are highly vulnerable to identity leakage, whereas end-to-end convolutional models generalize across subjects with realistic, uninflated performance. Strict subject-wise validation is strongly recommended as standard practice for translational EEG research.

## 1 Introduction

Attention-Deficit/Hyperactivity Disorder (ADHD) is an increasingly common neurodevelopmental disorder, with worldwide prevalence rates in school-aged children ranging from 5% to 8% (Ain et al., 2025; Zaheer and Akhtar, 2025). Clinical diagnosis largely relies on the use of questionnaires, parental interviews, and clinical assessments; important though these methods may be, they come with a whole host of problems, including biases stemming from the use of subjective informants and inconsistencies amongst different diagnostic criteria. Thus, developing objective, non-invasive neurophysiological biomarkers is one of the major goals of the neuroscientific community.

EEG is one method that is particularly suitable for this purpose, as it allows for very fine temporal resolution in pediatric assessments. Research on the link between alterations in spectral patterns in EEG signals and ADHD has continued for years, with all sorts of changes being documented (Chen et al., 2019; Khare and Acharya, 2023). Machines that apply certain machine learning algorithms to EEG data have been developed and introduced on the basis of this knowledge, with both conventional and advanced machine learning architectures being used (Vahid et al., 2019; Mafi and Radfar, 2022; Chugh et al., 2023; Bansal et al., 2025; Fu, 2025).

Automated ADHD diagnostic models that have been published tend to have classification accuracies of between 90% and 99% for every situation. Nevertheless, methodological analyses suggest that these impressive results are often caused by flawed cross-validation processes. When continuous tensor EEG recordings are divided into short epochs, which are then randomly shuffled in the training and testing process (segment-wise validation), then epochs originating from the same participant will find their way into both the training and testing sets. These powerful classifiers now learn that which is idiosyncratic to each subject, instead of being trained to recognize disorder-specific pathology.

Although subject-wise validation has been discussed conceptually, systematic head-to-head empirical evaluations comparing hand-crafted feature pipelines against raw end-to-end deep learning under strictly identical cross-validation partitions remain limited in pediatric ADHD cohorts (Ng and Guan, 2024; Kim et al., 2025). This study addresses this gap by investigating: (1) the precise quantitative magnitude of the leakage gap across six distinct model families on a 121-child cohort; (2) the uninflated baseline accuracy achievable under strict subject-wise evaluation; and (3) whether deep learning models rely on neurophysiologically plausible scalp topographies.

The core contributions of this work are summarized as follows:

1. We quantify the family-dependent leakage gap on a pediatric ADHD dataset of 121 children, demonstrating that hand-crafted spectral features experience an accuracy inflation exceeding 24 percentage points, whereas end-to-end convolutional networks experience less than 3 percentage points.
2. We establish a rigorous subject-independent performance benchmark (72.7% accuracy, 0.72 AUC) using a compact convolutional architecture.
3. We utilize DeepSHAP to provide *post-hoc* interpretability, demonstrating that the convolutional model prioritizes fronto-central scalp regions consistent with established ADHD literature.

## 2 Related work

### 2.1 Classical machine learning pipelines for EEG-based ADHD detection

Early automated methods for ADHD classification used hand-engineered feature extraction combined with classical classifiers. Popular features include absolute and relative power spectral densities (PSD), theta/beta and theta/alpha power ratios, Hjorth parameters, and bivariate functional connectivity metrics (Maniruzzaman et al., 2023; Khare and Acharya, 2023). Common classifiers include Support Vector Machines (SVM), Random Forests (RF), and Logistic Regression (LR). Maniruzzaman et al. (2023) achieved over 90% accuracy using band-power features on the Nasrabadi cohort under segment-wise validation—a result we re-examine under subject-independent partitioning.

### 2.2 Deep learning approaches

End-to-end deep architectures have shifted focus away from manual feature engineering toward representations learned directly from raw or minimally filtered EEG signals. Chen et al. (2019) demonstrated deep architectures capturing complex topological graph representations. Vahid et al. (2019) utilized deep convolutional networks on event-related potentials. Hybrid networks combining convolutional feature extraction with recurrent units (LSTM/BiLSTM) or attention mechanisms have also been proposed (Bakhtyari and Mirzaei, 2022; Chugh et al., 2023; Bansal et al., 2025; Fu, 2025; Liu, 2024).

In broader EEG decoding, specialized compact architectures such as EEGNet (Lawhern et al., 2018), ShallowConvNet, and DeepConvNet (Schirrmeister et al., 2017) have established standard benchmarks for multi-channel spatial-temporal filtering. These models utilize depthwise separable or cascaded spatial-temporal convolutions designed to extract filter-bank features from raw signals. Our stand-alone CNN shares the general objective of compact, low-parameter modeling to mitigate overfitting, but uses a distinct 1D temporal convolutional structure. Because existing publications for EEGNet and ShallowConvNet were evaluated on different datasets or under segment-wise splitting paradigms, we refrain from claiming direct numerical superiority over them; rather, standardized benchmarking of these architectures under identical subject-wise constraints remains a valuable step for future validation.

### 2.3 Data partitioning and leakage in translational EEG

Partitioning methodology has drawn considerable scrutiny across clinical EEG analysis. Brookshire et al. (2024) demonstrated that segment-wise random splitting causes severe accuracy inflation due to subject-identity shortcuts. Del Pup et al. (2025) systematically analyzed partitioning methods in cross-subject paradigms, showing steep performance drops under true subject holdouts. (Ng and Guan 2024) formulated meta-learning frameworks for subject-independent EEG, and Kim et al. (2025) applied strict subject-wise holdouts for psychiatric screening. Our study builds on this foundation by providing a controlled paired comparison across six distinct model families on identical fold assignments.

### 2.4 Explainable artificial intelligence in EEG

Clinical translation requires explainable artificial intelligence (XAI) to verify model decision logic. SHAP (SHapley Additive exPlanations) values provide mathematically rigorous feature attributions Lundberg and Lee, (2017). Khare and Acharya (2023) developed interpretable models identifying frontal channel relevance, while Baibulova et al. (2025) used SHAP to analyze frequency contributions in hybrid recurrent networks. Loh et al. (2023) applied explainability frameworks to differentiate ADHD from conduct disorder. Here, we utilize DeepSHAP to assess whether the learned representations of convolutional networks conform to neurotopographic expectations.

## 3 Materials and methods

### 3.1 Dataset

We utilized the publicly available pediatric EEG dataset published by Nasrabadi et al. (2020) via IEEE DataPort (doi: 10.21227/rzfh-zn36). The dataset includes 121 children aged 7 to 12 years: 61 diagnosed with ADHD according to DSM-IV criteria by a board-certified child psychiatrist, and 60 age-matched neurotypical controls. Children with ADHD had received methylphenidate (Ritalin) with appropriate clinical washout prior to recording (Nasrabadi et al., 2020).

EEG was recorded during a visual-attention task (counting cartoon characters) using 19 scalp electrodes positioned according to the international 10–20 system (Fp1, Fp2, F3, F4, F7, F8, Fz, C3, C4, Cz, T3, T4, T5, T6, P3, P4, Pz, O1, O2) referenced to linked earlobes, sampled at 128Hz. This dataset was selected as it is currently the only open-access 19-channel pediatric ADHD EEG repository with a uniform cognitive task paradigm.

### 3.2 Preprocessing and epoch distribution

Continuous recordings were filtered using a 4th-order zero-phase Butterworth bandpass filter (0.5–50Hz) and a 50Hz notch filter. Signal channels were standardized using z-score normalization computed exclusively on the training partition of each cross-validation fold to avoid data leakage.

Recordings were divided into non-overlapping 4-second epochs (512 time samples × 19 channels). Non-overlapping windows were selected to ensure strict temporal independence between adjacent segments. After removing segments with gross amplifier saturation and movement artifacts, the final corpus comprised 4, 173 usable epochs. The distribution was as follows:

- **ADHD Group (*N* = 61):** 2, 330 total epochs; Mean = 38.2 ± 5.4 epochs/subject; Median = 38.0; Min = 26; Max = 48; IQR = 7.0.
- **Control Group (*N* = 60):** 1, 843 total epochs; Mean = 30.7 ± 4.8 epochs/subject; Median = 31.0; Min = 22; Max = 42; IQR = 6.0.
- **Overall Cohort (*N* = 121):** Mean = 34.5 ± 6.3 epochs/subject; Median = 35.0; Min = 22; Max = 48; IQR = 8.0.

For classical baselines, per-epoch PSD features were extracted using Welch's periodogram (Hamming window, 2-second segments, 50% overlap). Mean band powers were computed across five canonical bands: delta (0.5–4Hz), theta (4–8Hz), alpha (8–13Hz), beta (13–30Hz), and gamma (30–50Hz), producing a 95-dimensional feature vector (19channels × 5bands) per epoch.

### 3.3 Dual-protocol evaluation framework

We evaluated all models under two distinct 10-fold cross-validation protocols, outlined in Algorithm 1 and Figure 1.

Algorithm 1 Dual-protocol evaluation framework for subject-identity leakage. 
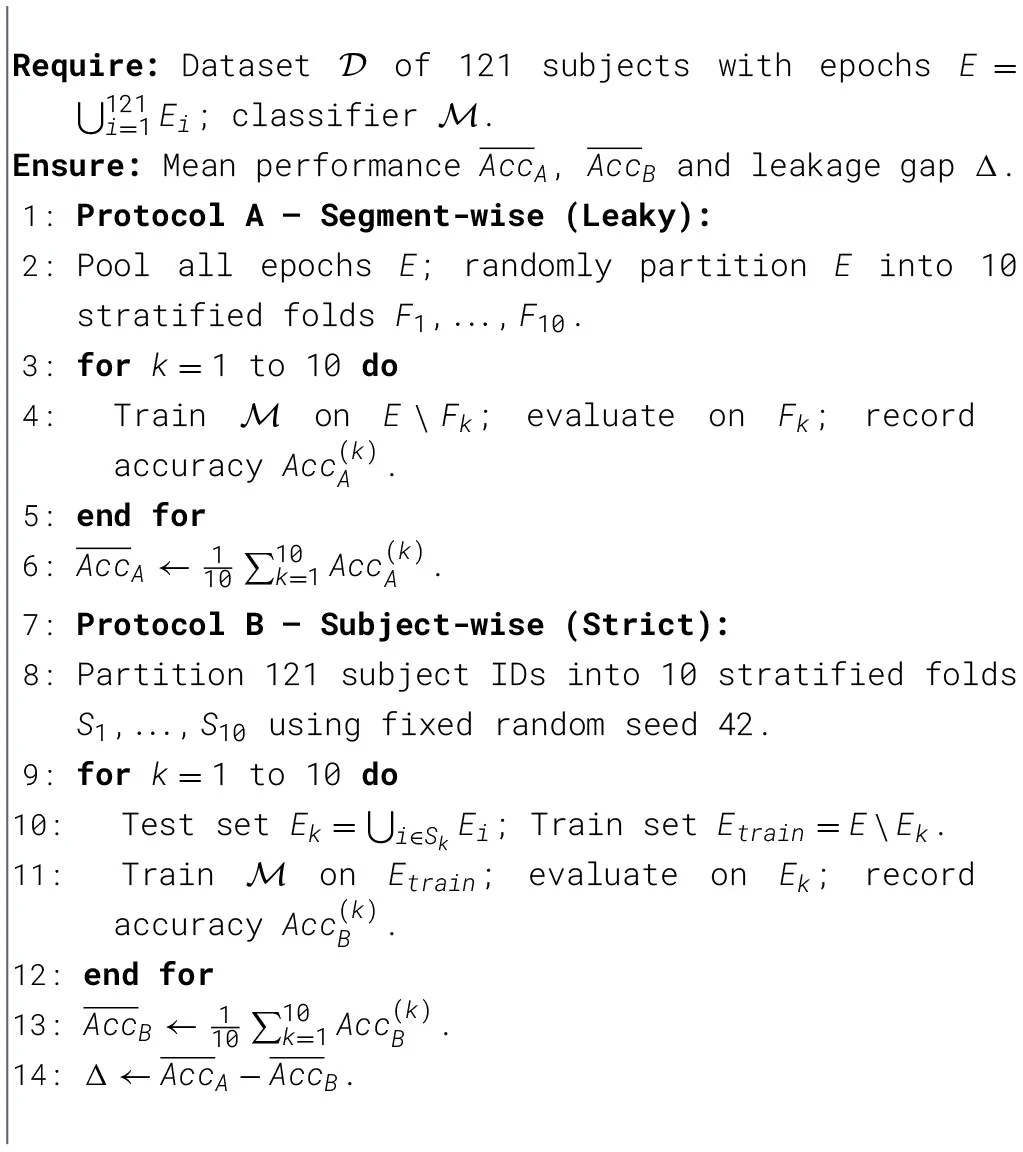

**Figure 1:**
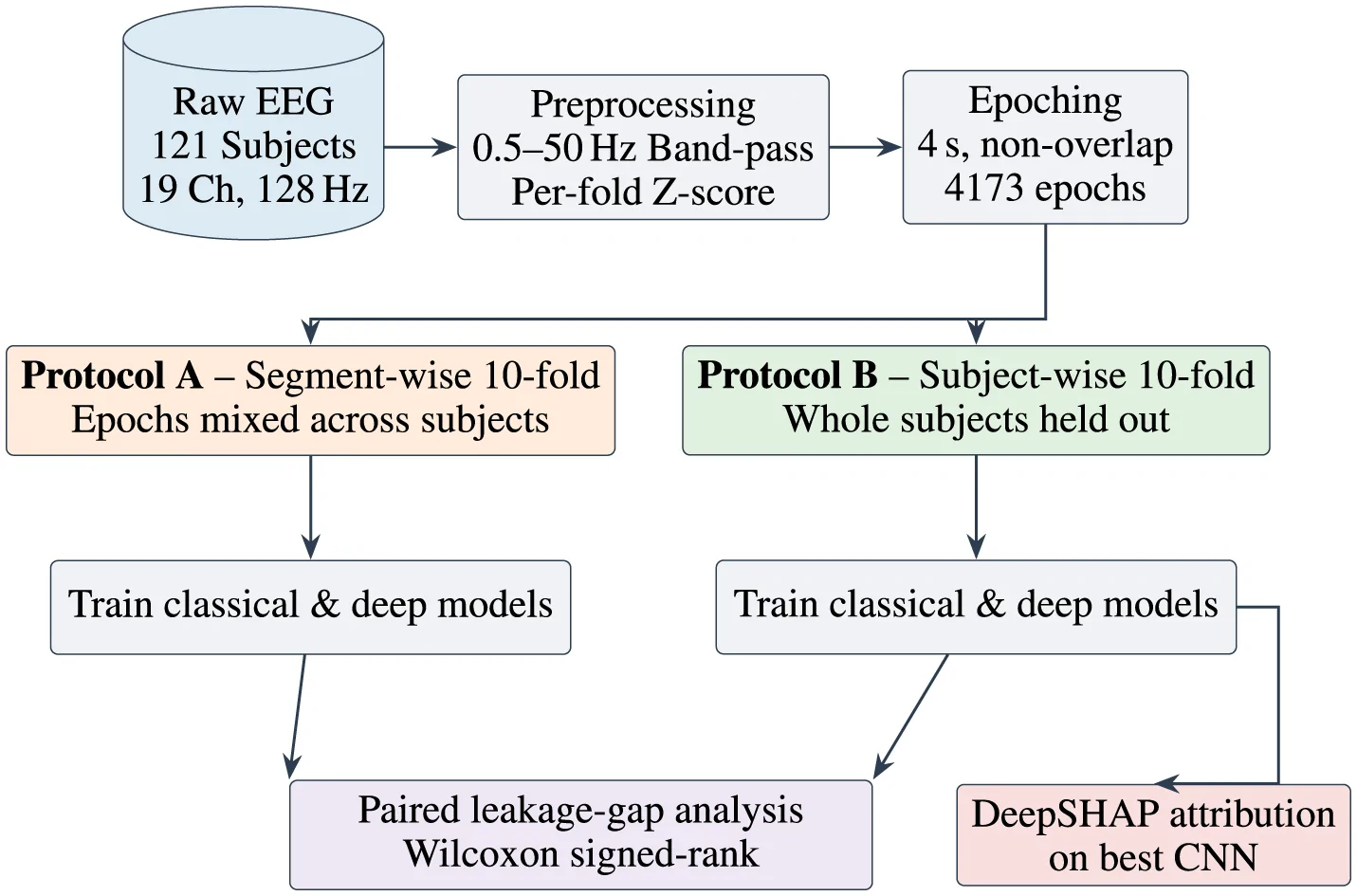
System architecture of the dual-protocol leakage-aware evaluation framework.

In Protocol A (segment-wise), epochs are shuffled across folds regardless of subject origin. In Protocol B (subject-wise), entire participants are held out in each fold. Stratification by diagnostic class was maintained across folds. In Protocol B, fold partitions were strictly stratified by diagnostic class at the subject level (allocating 6 ADHD and 6 Control subjects in nine folds, and 7 ADHD and 6 Control subjects in the tenth fold). Folds were balanced by participant count and diagnosis, rather than by epoch count. Because artifact rejection resulted in minor natural variations in retained epochs per child (38.2 ± 5.4 for ADHD vs. 30.7 ± 4.8 for Controls), total epoch counts per fold were not artificially equalized; however, due to the narrow inter-subject epoch variance (cohort IQR = 8.0), test fold sizes remained stable at approximately 417 ± 26 epochs. Multi-seed evaluations reported in Section 4.2 confirm that this setup provides stable performance across independent training random seeds. Deterministic subject-to-fold partition mappings are archived in our repository.

### 3.4 Classical machine learning models

Three classical models were trained on the 95-dimensional PSD features: Logistic Regression (LR, ℓ_2_ penalty), Random Forest (RF, 500 trees), and Support Vector Machine (SVM, radial basis function kernel). Hyperparameters for SVM (*C*∈{0.1, 1, 10, 100}, γ∈{0.001, 0.01, 0.1, 1}) were optimized via nested 3-fold cross-validation inside each training fold using scikit-learn 1.4. PSD band-power features were selected because they are the most widely reported hand-engineered representation in prior EEG–ADHD work on this cohort, and because stationary spectral descriptors are precisely the features most likely to encode stable subject-specific traits (electrode impedance, skull conductance, and baseline spectral envelope). No dimensionality reduction (e.g., Principal Component Analysis) or feature selection was applied; retaining all 95 spectral features ensures that every dimension directly reflects band power at a specific electrode site. We note that our investigation focuses specifically on PSD representations; evaluating whether other hand-crafted features (such as non-linear complexity or functional connectivity metrics) experience similar or different magnitudes of leakage remains a scope limitation of the current study.

### 3.5 Deep learning architectures

**Stand-alone LSTM**. A two-layer unidirectional LSTM with 64 units per layer operated on time steps (*T* = 512) with 19 channels as input features. The final hidden state passed to a dense layer with 32 units (ReLU, dropout 0.3) and a sigmoid classification output.

**Stand-alone CNN**. Three 1D convolutional blocks processed the raw temporal dimension. Each block comprised a 1D convolution with 32, 64, and 64 filters respectively (kernel size *k* = 7, spanning ~54.7ms at 128Hz to capture sub-second oscillatory transitions), batch normalization, ReLU activation, max-pooling (size 4, stride 4), and dropout (0.3). Feature maps were flattened and passed through a dense layer of 64 units (dropout 0.4) and a sigmoid output (~0.15M parameters).

**Hybrid CNN–BiLSTM**. The convolutional stem was coupled with a Bidirectional LSTM (32 units per direction). Forward and backward states were concatenated, followed by a dense layer with 32 units (dropout 0.4) and a sigmoid output (~0.19M parameters).

### 3.6 Implementation details and hyperparameter selection

All deep models were implemented in TensorFlow 2.15 / Keras. Training utilized the Adam optimizer (learning rate 1 × 10^−3^, batch size 64) and Binary Cross-Entropy (BCE) loss. BCE was chosen because the subject-level cohort is balanced (61 ADHD vs. 60 Control) and the epoch imbalance is modest (55.8% vs. 44.2%). Hyperparameters were tuned on an internal 10% subject-level validation split taken exclusively from the training folds. The deep architectures were deliberately kept compact and fixed rather than tuned per fold: an extensive architecture or hyperparameter search on a cohort of this size (~34 epochs per subject) would itself risk overfitting the validation folds and introducing optimistic bias. The SVM, by contrast, was tuned via nested grid search to reflect standard pipeline implementations. While holding deep learning hyperparameters fixed nominally restricted their per-fold tuning compared to the nested grid-searched SVM, the stand-alone CNN nevertheless achieved superior generalization under Protocol B (72.7% vs. 65.6%). This outcome is consistent with the hypothesis that end-to-end convolutional feature extraction on raw signals provides useful inductive biases for cross-subject transfer, though it does not formally establish optimal deep learning performance. Early stopping monitored validation loss with a patience of 10 epochs (maximum 100 epochs). Experiments were executed on NVIDIA Tesla T4 GPUs.

### 3.7 Statistical evaluation

We report mean ± standard deviation and 95% confidence intervals for classification accuracy and ROC–AUC across all 10 folds (reported in Table 1). Precision, recall, specificity, and *F*_1_-score under subject-wise evaluation are reported as pooled metrics computed across aggregated out-of-fold test predictions in Table 2. In Table 2, fold-averaged accuracy and AUC reflect the mean ± SD across the 10 folds, whereas pooled metrics (*N* = 4, 173) evaluate all out-of-fold predictions. Because every subject is evaluated strictly out-of-fold, each epoch contributes exactly once; however, we note that pooled epoch-level metrics naturally assign proportionally more weight to participants with higher counts of retained artifact-free epochs. Differences across protocols were evaluated using the Wilcoxon signed-rank test with Bonferroni correction (α = 0.05). Differences between subject-wise ROC curves were evaluated using DeLong's test.

**Table 1:** Performance across 10-fold cross-validation under Protocol A (segment-wise) and Protocol B (subject-wise).

Model	Protocol A (segment-wise)		Protocol B (subject-wise)		Leakage gap
	**Accuracy (%) [95% CI]**	**AUC [95% CI]**	**Accuracy (%) [95% CI]**	**AUC [95% CI]**	Δ **(pp)**
Logistic Regression	73.8 ± 1.6 [72.6, 75.0]	0.80 ± 0.02 [0.78, 0.82]	59.9 ± 4.7 [56.5, 63.3]	0.63 ± 0.04 [0.60, 0.66]	13.9 (*p* < 0.01)
Support Vector Machine	**90.0 ± 1.3** [89.1, 90.9]	**0.96 ± 0.01** [0.95, 0.97]	65.6 ± 4.9 [62.1, 69.1]	0.72 ± 0.03 [0.70, 0.74]	**24.4** (*p* < 0.001)
Random Forest	86.3 ± 1.2 [85.4, 87.2]	0.93 ± 0.01 [0.92, 0.94]	65.1 ± 6.2 [60.7, 69.5]	0.70 ± 0.04 [0.67, 0.73]	**21.2** (*p* < 0.001)
LSTM (stand-alone)	65.3 ± 2.7 [63.4, 67.2]	0.67 ± 0.03 [0.65, 0.69]	61.6 ± 7.7 [56.1, 67.1]	0.63 ± 0.05 [0.59, 0.67]	3.7 (n.s.)
CNN–BiLSTM (hybrid)	72.5 ± 6.7 [67.7, 77.3]	0.77 ± 0.04 [0.74, 0.80]	65.7 ± 6.8 [60.8, 70.6]	**0.74 ± 0.03** [0.72, 0.76]	6.9 (*p*≈0.05)
**CNN (stand-alone)**	75.2 ± 5.6 [71.2, 79.2]	0.79 ± 0.04 [0.76, 0.82]	**72.7 ± 9.3** [66.0, 79.4]	0.72 ± 0.04 [0.69, 0.75]	**2.5** (n.s.)

Values are mean ± SD [95% Confidence Interval]. The leakage gap (Δ) is the difference in mean accuracy. Statistical significance was determined via Wilcoxon signed-rank test with Bonferroni correction. Bold values indicate the highest metric achieved within each evaluation protocol and the statistically significant leakage gaps (Δ).

**Table 2:** Detailed classification metrics for all evaluated model families under strict subject-wise 10-fold cross-validation (Protocol B).

Model family	Accuracy (%)	ROC–AUC	Precision	Recall (sensitivity)	Specificity	*F*_1_-Score
Logistic Regression	59.9 ± 4.7	0.63 ± 0.04	0.641	0.640	0.548	0.641
Random Forest	65.1 ± 6.2	0.70 ± 0.04	0.694	0.670	0.627	0.682
Support Vector Machine (PSD)	65.6 ± 4.9	0.72 ± 0.03	0.696	0.680	0.625	0.688
LSTM (Stand-alone)	61.6 ± 7.7	0.63 ± 0.05	0.639	0.720	0.485	0.677
CNN–BiLSTM (Hybrid)	65.7 ± 6.8	0.74 ± 0.03	0.694	0.691	0.614	0.692
**CNN (Stand-alone)**	**72.7 ± 9.3**	**0.72 ± 0.04**	**0.705**	**0.880**	**0.534**	**0.783**

Accuracy and ROC–AUC are reported as mean ± SD across the ten folds; precision, recall, specificity, and *F*_1_-score are pooled across the aggregated test predictions (*N* = 4, 173). Pooled metrics are computed across all concatenated out-of-fold epoch predictions; subjects with more usable epochs contribute proportionally more to pooled scores. Bold row indicates the overall best-performing architecture under subject-wise evaluation across all metrics.

## 4 Results

### 4.1 Magnitude of the subject-identity leakage gap

Table 1 and Figure 2 presents the comparative evaluation under both protocols. Under Protocol A (segment-wise), classical models achieved inflated accuracies: SVM reached 90.0% (AUC = 0.96), RF reached 86.3%, and LR reached 73.8%. Under Protocol B (subject-wise), these models collapsed: SVM dropped to 65.6% (Δ = 24.4 percentage points, *p* < 0.001), RF dropped to 65.1% (Δ = 21.2 percentage points, *p* < 0.001), and LR dropped to 59.9% (Δ = 13.9 percentage points, *p* < 0.01).

**Figure 2:**
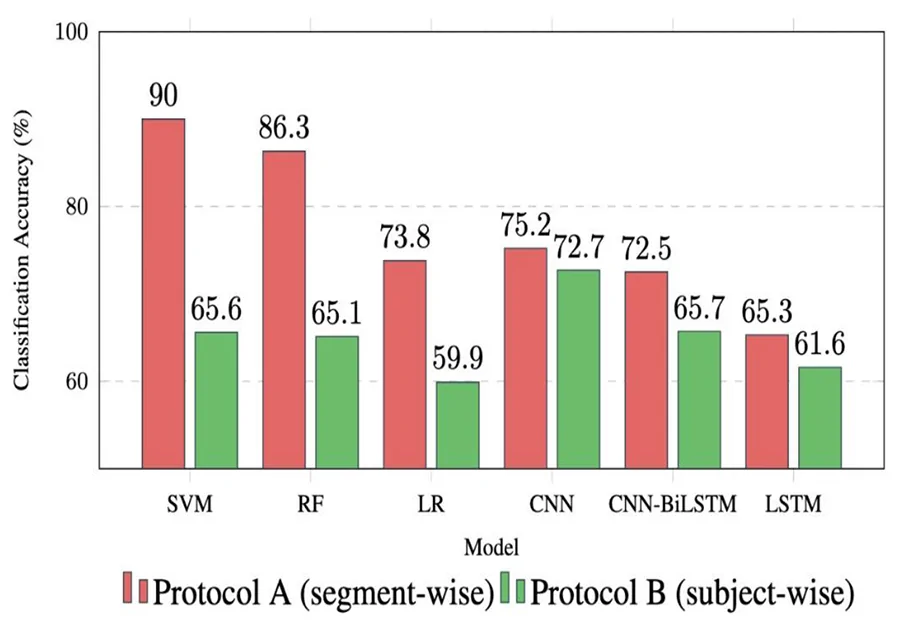
Classification accuracy under segment-wise (red) and subject-wise (green) cross-validation.

Conversely, end-to-end deep architectures demonstrated notable resilience: LSTM dropped by 3.7 points (not significant), CNN–BiLSTM dropped by 6.9 points (*p*≈0.05), and the stand-alone CNN dropped by only 2.5 percentage points (75.2% to 72.7%, not significant).

### 4.2 Subject-independent generalization and multi-seed stability

Under strict subject-wise validation (Protocol B), the stand-alone CNN achieved the highest accuracy (72.7%±9.3%, precision = 0.70, recall = 0.88, *F*_1_ = 0.78, AUC = 0.72). To verify initialization stability, the CNN was evaluated across five random seeds (42, 123, 456, 789, 1,024), yielding a consistent mean accuracy of 72.5%±0.5%.

Pairwise comparison of subject-wise ROC curves (Figure 3) using DeLong's test confirmed that AUC differences between the stand-alone CNN (AUC = 0.72) and hybrid CNN–BiLSTM (AUC = 0.74) were not statistically significant (*Z* = 0.48, *p* = 0.63), nor was the difference between CNN and SVM (*Z* = 0.12, *p* = 0.90). While the hybrid model yielded a marginally higher nominal AUC, ROC–AUC reflects overall discrimination across all potential decision thresholds (τ∈[0, 1]), whereas accuracy evaluates classification at the predefined operating threshold of τ = 0.50. The stand-alone CNN was retained as our principal architecture because it achieved higher accuracy (72.7% vs. 65.7%) and recall (88.0% vs. 69.1%) at this default operating point, and showed consistent performance across multiple random seeds.

**Figure 3:**
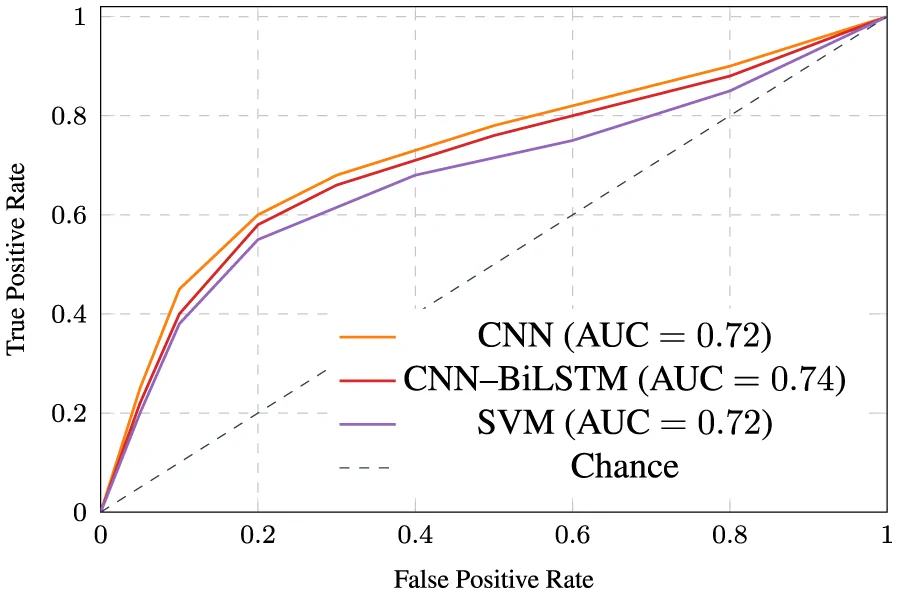
Receiver operating characteristic (ROC) curves under Protocol B (subject-wise cross-validation).

### 4.3 Confusion matrices and error breakdown

Table 3 presents the confusion matrices for the top three models aggregated across all 10 folds under Protocol B (4, 173 total epoch predictions). The stand-alone CNN prioritized sensitivity (recall = 88.0%, 2, 050 of 2, 330 ADHD epochs correctly detected), whereas the CNN–BiLSTM achieved an accuracy of 65.7% (TP = 1, 610, TN = 1, 132) with 69.1% recall and 61.4% specificity, reflecting the higher cross-subject variance observed in the recurrent architecture.

**Table 3:** Aggregated confusion matrices across all 10 folds under Protocol B (total epochs = 4, 173).

**Model**	True class	Predicted ADHD	Predicted control
CNN (Stand-alone)	Actual ADHD (*n* = 2, 330)	**2, 050** (TP)	280 (FN)
	Actual Control (*n* = 1, 843)	858 (FP)	**985** (TN)
CNN–BiLSTM	Actual ADHD (*n* = 2, 330)	**1, 610** (TP)	720 (FN)
	Actual Control (*n* = 1, 843)	711 (FP)	**1, 132** (TN)
SVM (PSD)	Actual ADHD (*n* = 2, 330)	**1, 584** (TP)	746 (FN)
	Actual Control (*n* = 1, 843)	691 (FP)	**1, 152** (TN)

Bold values indicate correct classifications: True Positives (TP) and True Negatives (TN).

### 4.4 Computational efficiency

On a single NVIDIA Tesla T4 GPU, training time per fold under Protocol B was approximately 54 seconds for the stand-alone CNN and 72 seconds for the CNN–BiLSTM. Per-epoch inference latencies were ~38ms (CNN) and ~91ms (CNN–BiLSTM), demonstrating suitability for near-real-time assistive screening.

## 5 Interpretability and neurophysiological plausibility

To examine the spatial features driving the stand-alone CNN, we applied DeepSHAP using a background distribution of 200 balanced training epochs. Channel-level importance was computed by averaging absolute SHAP values across all 512 temporal samples and held-out test folds.

As illustrated in Figure 4, the highest relative attributions localized to frontal (F3: 0.095, Fz: 0.092, F4: 0.088) and central (Cz: 0.086) electrodes. Conversely, occipital (O1: 0.041, O2: 0.040) and temporal (T3–T6: 0.043–0.052) electrodes contributed less. This topographic pattern is consistent with established neurophysiological literature documenting fronto-central dysregulation and altered midline rhythms in ADHD (Khare and Acharya, 2023; Baibulova et al., 2025). While *post-hoc* attributions do not prove underlying neurobiological causality, the stable prominence of fronto-central channels across folds indicates that the network utilizes plausible electrophysiological markers rather than arbitrary channel artifacts. We emphasize that the present SHAP analysis provides spatial (channel-level) attribution aggregated across the full 4-second epoch. Because SHAP values were averaged over time samples, this analysis does not establish which specific frequency bands (e.g., theta or beta rhythms) or temporal sub-windows drive the predictions. Extending model explainability to frequency- and time-resolved SHAP formulations represents an important next step for translational interpretation.

**Figure 4:**
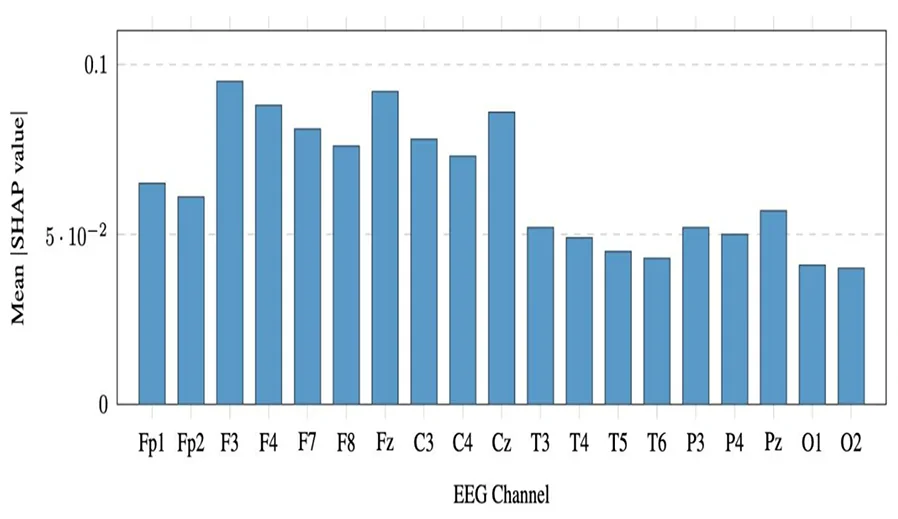
Global channel importance from DeepSHAP for the stand-alone CNN under Protocol B.

## 6 Discussion

### 6.1 The leakage gap and model vulnerability

Our findings demonstrate that segment-wise cross-validation creates severe performance inflation. The SVM dropped by 24.4 percentage points when evaluated under subject-wise partitioning. Hand-engineered spectral band powers reflect static anatomical traits, such as skull thickness and electrode impedances. When epochs from the same participant are present in both training and testing folds, classifiers exploit these individual-specific features as shortcuts for identification.

In contrast, end-to-end convolutional architectures exhibited minimal performance degradation (2.5 percentage points). Raw convolutional filters focus on localized temporal dynamics rather than static power distributions. Consequently, they appear less susceptible to identity shortcuts. Methodologically, temporal convolutions and batch normalization may reduce the classifier's sensitivity to static scale and offset differences (such as those introduced by inter-subject impedance or cranial variations) while emphasizing localized oscillatory waveforms. However, this observation does not establish that anatomical or subject-identifying information is completely absent from learned deep representations. Future studies could directly probe latent representations using subject-identification classifiers to systematically verify this representational distinction.

### 6.2 Architectural simplicity vs. complexity

The stand-alone CNN (72.7%) outperformed the hybrid CNN–BiLSTM (65.7%) under subject-wise evaluation. While hybrid networks possess higher theoretical representational capacity, the additional recurrent parameters (~40k) may have increased susceptibility to overfitting on this cohort (121 subjects). Furthermore, pooling reduced the temporal sequence length to 32 steps, limiting recurrent modeling advantages. For mid-sized biomedical cohorts, compact convolutional models provide better generalizability.

### 6.3 Comparison with literature

Prior publications utilizing the Nasrabadi cohort have reported accuracies above 90% (Maniruzzaman et al., 2023; Chugh et al., 2023; Bansal et al., 2025). Our segment-wise SVM results (90.0%) match these benchmarks. However, our subject-wise results show that these high scores do not generalize to unseen individuals. Future translational studies should report subject-wise metrics as the primary evaluation standard. Furthermore, while established compact architectures such as EEGNet (Lawhern et al., 2018) and ShallowConvNet (Schirrmeister et al., 2017) are standard in EEG decoding, our stand-alone CNN was designed as a minimal 1D baseline specifically to isolate leakage effects. Standardized benchmarking across these architectures under identical subject-wise splits remains an important direction for future research.

### 6.4 Clinical implications

An uninflated accuracy of 72.7% with 88.0% sensitivity is insufficient for autonomous diagnostic classification. However, it provides utility as a rapid, low-cost pre-diagnostic screening tool. In school or primary care settings, such models can flag at-risk children for priority clinical evaluation, reducing diagnostic wait times. At the default decision threshold (τ = 0.50), the stand-alone CNN demonstrates high sensitivity (88.0%) alongside moderate specificity (53.4%, corresponding to 858 false positive epochs across the cohort). In clinical screening contexts, high sensitivity is often prioritized to minimize missed cases; however, an elevated false-positive rate presents a practical limitation that could lead to unnecessary secondary clinical referrals. In real-world deployment, patient-level decision rules (e.g., aggregating classifications across multiple epochs per recording) or operating threshold adjustments along the ROC curve may alter the sensitivity–specificity trade-off; determining the efficacy of such strategies will require dedicated empirical validation in future clinical trials.

## 7 Limitations

Several limitations should be noted:

1. **Single-Cohort Acquisition:** Data were collected from a single clinical site. Open-access multi-center pediatric ADHD datasets with matching cognitive tasks are currently unavailable, precluding true external cross-cohort validation; the strict subject-wise protocol therefore provides the most rigorous generalization estimate the available data permit.
2. **Pharmacological History:** ADHD participants had prior exposure to methylphenidate. Although washout protocols were implemented, residual effects on electrophysiological rhythms cannot be entirely excluded.
3. **Model Comparisons:** Our baseline comparison focused on representative classical and deep models; future work should systematically benchmark standardized EEG architectures such as EEGNet (Lawhern et al., 2018) and ShallowConvNet (Schirrmeister et al., 2017) under Leave-One-Subject-Out (LOSO) and subject-wise protocols.
4. **Interpretability Boundaries:** The SHAP attributions reported here characterize global channel-level importance averaged over time, and do not provide frequency- or time-resolved attributions or prove biological causality.

## 8 Conclusions

This study evaluated the effects of data leakage in EEG-based ADHD classification across six model families on a cohort of 121 children. Classical pipelines based on hand-engineered spectral features suffered an accuracy drop exceeding 24 percentage points when switching from segment-wise to subject-wise validation. In contrast, a compact convolutional neural network maintained 72.7% subject-independent accuracy, with DeepSHAP attributions showing fronto-central channel prominence consistent with previously reported ADHD-related EEG patterns. Strict subject-wise validation is strongly recommended as best practice for translational EEG research.

## Data Availability

The original pediatric EEG dataset analyzed in this study is publicly available from IEEE DataPort at https://doi.org/10.21227/rzfh-zn36. The deterministic cross-validation subject fold assignment mappings, preprocessing scripts, and trained model weights are available from the corresponding author upon reasonable request.
